# Iodine

**DOI:** 10.1016/j.advnut.2024.100168

**Published:** 2024-01-05

**Authors:** Louise Brough, Sheila Skeaff

**Affiliations:** 1 School of Food and Advanced Technology, College of Sciences, Massey University, Palmerston North, New Zealand; 2 Department of Human Nutrition, University of Otago, Dunedin, New Zealand

**Keywords:** iodine deficiency disorders, iodized salt, thyroid function, thyroid hormones, median urinary iodine concentration

## Iodine [1]

The micronutrient iodine is essential for thyroid function. Iodine is a constituent of the thyroid hormones, thyroxine (tetraiodothyronine, T4), and triiodothyronine (T3) that control metabolism, growth, and development [2].

Dietary iodine is absorbed by the gut and actively trapped by the thyroid gland. Iodine is bound to a tyrosine residue of thyroglobulin to produce monoiodotyrosine and diiodotyrosine residues. These can then coalesce to produce T3 and T4 [3]. T3 is the active hormone and can also be produced by the action of de-iodinase hormones on T4. The adult human body contains around 15–20 mg iodine of which the thyroid contains 70%–80%. Excess iodine is excreted via the kidneys and 90% of ingested iodine is excreted via urine, and there are also small losses via the feces and sweat from the skin.

Thyroid hormone concentrations are regulated by the hypothalamic-pituitary-thyroid axis. Low blood concentrations of thyroid hormones trigger the hypothalamus to secrete thyrotropin-releasing hormone (TRH), which in turn causes the anterior pituitary gland to secrete thyroid-stimulating hormone (TSH or thyrotropin). TSH then induces thyroid activity including trapping of iodine and thyroid hormone synthesis and secretion. Conversely, high blood iodine concentrations lead to downregulation of TRH and TSH. Excessive blood iodine concentrations can result in the thyroid gland effectively shutting down and an acute decrease of thyroid hormone synthesis, known as the Wolff-Chaikoff effect [4].

## Deficiency

Although the most commonly known symptom is goiter, iodine deficiency can cause a range of other adverse consequences on growth and development that are collectively referred to as “iodine deficiency disorders” or IDDs. Iodine deficiency is categorized as mild, moderate, or severe, depending on the nature of the consequence and its impact on quality of life; Table 1 is adapted from [5] and takes into consideration the severity of iodine deficiency. For example, a pregnant female who is severely iodine deficient can give birth to a child with congenital iodine deficiency syndrome or cretinism, which is characterized by profound irreversible mental impairment, stunting, deaf mutism, squint, and other physical abnormalities: consequently, this IDD is severe. In contrast, a healthy adult can become mildly iodine deficient and develop a goiter, which could be reversed by increasing the iodine content of the diet with no long-lasting effects: consequently, this IDD is mild.

**TABLE 1:**
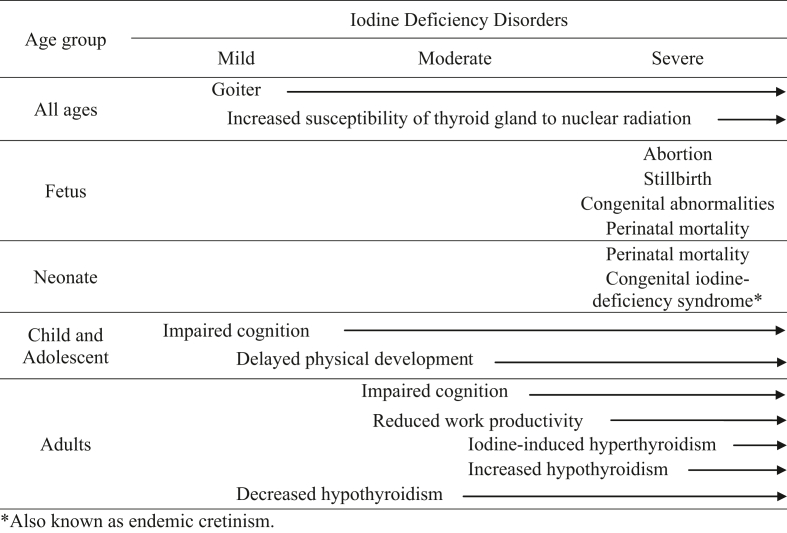
Consequences of iodine deficiency, by age group and severity.

IDDs occur when a lack of iodine causes thyroid hormone production to decrease. Because thyroid hormone is required for normal brain development and growth, an inadequate amount of thyroid hormone can be particularly detrimental at some stages of the life cycle, such as pregnancy and childhood. Given the serious consequences of iodine deficiency in infants and children, a global effort was launched in the 1990s to improve iodine intakes with a focus on iodized salt. In 1993, of a 121 countries that measured iodine status, only 8 (7%) countries had adequate iodine status [6]. In 2021, of the 152 countries studied, 128 (84%) countries had adequate iodine status, a result hailed as a “public health success story” [7]. Of interest in 2021 is that 13 of the 152 countries had an excessive iodine intake, highlighting the need for routine surveillance of iodine status, and if required, adjusting the iodine content of salt.

## Diet Recommendations

The Dietary Reference Intakes (DRI) for iodine for the United States and Canada were last updated in 2006 (Table 2) [8].

**TABLE 2:** United States Dietary Reference Intakes for iodine

	United States National Academy of Medicine^1^			
Life stage	Estimated average requirement (EAR) (μg/d)	Recommended dietary allowance (RDA) (μg/d)	Adequate intake (AI) (μg/d)	Tolerable upper level of intake (UL) (μg/d)
0–6 mo			110	Insufficient data to determine
7–12 mo			130	Insufficient data to determine
1–3 y	65	90		200
4–8 y	65	90		300
9–13 y (boys)	73	120		600
9–13 y (girls)	95	150		600
14–18 y (boys)	73	120		900
14–18 y (girls)	95	150		900
Males ≥19 y	95	150		1100
Females ≥19 y	95	150		1100
Pregnancy	160	220		900 (14–18 y) 1100 (≥19 y)
Lactation	209	290		900 (14–18 y) 1100 (≥19 y)

1 National Academy of Medicine [8].

## Food Sources

The iodine content of foods is influenced by the environment in which it has grown. Because seawater has a relatively high iodine concentration (50 μg/L) [3], marine foods, such as fish, seafood, and seaweed, tend to be the best natural sources. The iodine content of plants and animals depends on the iodine content of the soil, which is highly variable globally. To prevent deficiency, iodized salt is available in many countries and, in some countries, other staple foods are fortified with iodized salt (for example, bread in Australia and New Zealand). Although dairy products contain iodine, the amount varies depending on cattle feed supplements, iodized salt licks, and the use of iodophors as cleaning agents. In the United States, bread is not naturally high in iodine but some manufacturers use iodate salts as dough conditioners. In recent years, there has been a shift to consuming plant-based milks (such as oat, soy, and almond) that contain little iodine, which could lead to lower iodine intakes from milk, if other iodine sources are not consumed. Likewise, the recent trend for low carbohydrate diets is of concern in countries where bread is a significant contributor to iodine.

## Clinical Uses

Disinfectants containing iodine are used in clinical practice. A nuclear emergency event can lead to high levels of radioactive iodine that can be taken up by the thyroid gland. A high dose of potassium iodate (130 mg for adults) is recommended immediately after such an event to protect the thyroid gland from radiation poisoning.

## Toxicity

The thyroid is protected to some extent from transient iodine excess via the Wolff-Chaikoff effect. However, continued exposure to excess iodine can result in hypothyroidism, goiter, and thyroid cancer. A tolerable upper intake level is provided by the United States DRI for each population group (Table 2) [8], although these do not apply to those receiving iodine under medical supervision.

## Recent Research

Because severe iodine deficiency is now rare, attention has shifted to the effects of mild-to-moderate iodine deficiency on health and the consequences of excessive iodine intakes. The cutoff of a median urinary iodine concentration (mUIC) ≥100 μg/L to indicate adequate iodine status is now used for all population groups except pregnant females, but was devised for school-age children with a 24-h urine volume of 1 L [9]. The cutoff of mUIC ≥150 μg/L to indicate adequate iodine status in pregnant females aligns with the Recommended Dietary Allowance for iodine, which is set high to meet the iodine requirements of 97%–98% of pregnant females. Given this, the thresholds for mUIC used to define iodine status in populations need to be re-evaluated. Although a number of epidemiological studies have found a positive association between iodine status in pregnancy and cognition in children, the only randomized trial found no difference in neurodevelopment of children born to mothers who were mildly iodine deficient in pregnancy compared with mothers who were iodine sufficient [10]. More trials are required to elucidate the effects of mild iodine deficiency in pregnancy. Given the success of iodized salt, there are an increasing number of countries that have iodine excess. Little is known about the long-term consequences of a high intake of iodine, requiring research to identify the short- and long-term effects of excessive iodine intakes.

## Author contributions

The authors’ responsibilities were as follows – LB, SS: wrote and contributed equally to the manuscript, and read and approved the final version.

## Conflict of interest

The authors report no conflicts of interest.

## Funding

The authors reported no funding received for this study.
